# The impact of urban–rural medical insurance integration on medical impoverishment: evidence from China

**DOI:** 10.1186/s12939-023-02063-6

**Published:** 2023-11-23

**Authors:** Jinkang Huo, Mingzheng Hu, Shaojie Li

**Affiliations:** 1 Bay Area International Business School, Beijing Normal University, Zhuhai, 519087 China; 2 School of Public Health, Peking University, 38 Xue Yuan Road, Haidian District, Beijing, 100191 China; 3 China Center for Health Development Studies, Peking University, 38 Xue Yuan Road, Haidian District, Beijing, 100191 China

**Keywords:** Urban, Rural, Medical insurance, Medical impoverishment, City policy evaluation, China

## Abstract

**Background:**

Financial protection is a key dimension of Universal Health Coverage (UHC), and social medical insurance is an effective measure to provide financial protection. The aim of this study is to examine the impact of urban–rural medical insurance integration on medical impoverishment in China.

**Methods:**

We collected the time of integration policy in 337 prefecture-level cities across China, combined with the longitudinal database of China Labor-force Dynamics Survey (CLDS) from 2012–2016, and used a difference-in-differences (DID) method with multiple time periods at the city level to study the effect of urban–rural medical insurance integration on the medical impoverishment. Besides, to explore the heterogeneity of policy effects across populations, we conducted subgroup analyses based on respondents' age, household registration, and whether they were rural–urban migrants.

**Findings:**

A total of 8,397 samples were included in the study. The integration policy has significantly reduced the incidence of medical impoverishment (average treatment effect on the treated (ATT) =  − 0.055, *p* < 0.05). Subgroup analysis showed that the impacts on medical impoverishment varied by age group, and the integration policy has more effect on older people than on younger people (ATT for age 15–34 =  − 0.018, *p* > 0.05; ATT for age 35–54 =  − 0.042, *p* < 0.05; ATT for age 55–64 =  − 0.163, *p* < 0.01). Moreover, the impacts also varied by household registration. The integration policy has a more significant impact on rural residents (ATT for rural =  − 0.067, *p* < 0.05) compared to urban residents (ATT for urban =  − 0.007, *p* > 0.05). Additionally, the policy has a bigger influence on rural–urban migrants (ATT for rural–urban migrated =  − 0.086, *p* < 0.05) than on those who have not migrated (ATT for rural–urban unmigrated =  − 0.071, *p* < 0.05).

**Conclusion:**

China's policy of integrating urban–rural medical insurance has been successful in reducing medical impoverishment, especially for older age, rural, and rural–urban migrated people. It can be speculated that the integrating policy may be adapted to other similar settings in developing countries to reduce medical impoverishment.

**Supplementary Information:**

The online version contains supplementary material available at 10.1186/s12939-023-02063-6.

## Introduction

Medical impoverishment is a term used to describe a situation in which health payments result in a non-poor household becoming poor after paying for health services and unable to cover subsistence spending [1]. Globally, approximately 100 million people are impoverished by the disease each year [2]. China, the world's largest developing country, also faces serious medical impoverishment problems, especially in its rural areas. Studies showed that health payments increased the total number of rural poor households by 44.3% in China [3].

Social medical insurance can reduce the risk of potentially large health expenditures faced by households, [4–7] can act as financial protection for poor households, [8] and is a common means of achieving universal health coverage in most low- and middle-income countries [2, 8–11]. China also established its own medical insurance system. In 2002 and 2007, the Chinese government established the New Rural Cooperative Medical Scheme (NCMS) and the Urban Resident Basic Medical Insurance (URBMI), covering rural residents and the urban population, respectively. In 2011, these two medical insurance schemes together covered 78% of the Chinese population [12].

Despite the high level of population coverage that has been achieved, there are still certain shortcomings in this social medical insurance system. First, the benefit packages of the two medical insurance systems differ significantly. Since URBMI covers more medical services, it provides a higher level of financial protection than NCMS [13, 14]. Second, there are difficulties in medical insurance reimbursement for migrant populations. Since the type of medical insurance scheme available to each individual depends on the household registration location, farmers who move to urban areas for work or residence are usually not eligible for URBMI, and remain enrolled in NCMS [15]. However, benefits such as medical reimbursement of NCMS are not available across counties, [16] rural-to-urban migrants in urban areas is difficult to obtain health insurance reimbursement [13]. Thus, the inadequate NCMS and URBMI insurance systems exacerbate the inequalities between urban and rural health care [17–20] and are ineffective in reducing the incidence of medical impoverishment [17, 21, 22].

To address these issues, China has carried out the policy of urban–rural medical insurance integration, integrated the URBMI and NCMS, and conducted a series of reforms such as erasing the gap between urban and rural medical insurance benefits and improving medical insurance benefits [23]. The integrated medical insurance system is called Urban and Rural Resident Basic Medical Insurance (URRBMI). Since 2009, the urban and rural health insurance integration policy has gradually started to be implemented year by year in some cities. This integration policy was largely completed after early 2016, when the Chinese central government announced the unified completion of the integration of NCMS and URBMI nationwide [24].

Studies have now shown that the incidence of medical impoverishment decreased from 7.39% to 5.14% between 2010 and 2016 [20]. However, we are not certain that these decreased effects are brought about by the urban–rural medical insurance integration policy. Previous studies on the impact of urban–rural health insurance integration on medical impoverishment are very limited. Several existing studies used cross-sectional data or provincial-level data to prove the association between integrating policies and catastrophic health expenditure or financial risk protection, but few studies provided an analysis using longitudinal and city-level data, and few of them conducted subgroup analyses across populations. In order to critically assess whether the integration policy can reduce the incidence of medical impoverishment, this study collected the time of integration policy in 337 prefecture-level cities across China, combined with the CLDS database from 2012–2016, used a difference-in-differences (DID) method with multiple time periods to study the effect of urban–rural medical insurance integration on the medical impoverishment, and further analyzed population heterogeneity.

## Methods

### Data sources

Our data come from individual-level social survey databases and municipal-level databases. First, the individual-level panel data are obtained from the CLDS database, which is designed and implemented by the Center for Social Survey of Sun Yat-sen University, and has been conducted every two years since 2012, with a sample covering 29 provincial-level administrative regions in China (excluding Hong Kong, Macao, Taiwan, Tibet, and Hainan). The CLDS database uses a multi-stage, multi-level, probability sampling methodology proportional to the size of the labor force to make it nationally representative. The CLDS database was ethically approved by the Biomedical Ethics Committee of Sun Yat-sen University. We used the data obtained from the CLDS surveys in 2012, 2014, and 2016, and we saved the sample aged 15–64 years because the CLDS is a survey that focuses on the labor force sample and there are many outliers and missing values in the sample that are not in this age range.

Second, in terms of city-level data, as shown in Figure S1 of Supplemental Figure, we collected the time of integration policy of each prefecture-level city, based on public policy documents available on legal websites or on the websites of different city governments. These policy documents are issued and made public by the government and record the time when the urban and rural residents' medical insurance system was officially in operation. In total, we collected integration completion times for 337 prefecture-level administrative regions (i.e., all prefecture-level administrative regions in China). In addition, we also obtained data on city-level control variables through the *China City Statistical Yearbook*. Finally, we merged the individual-level data with the city-level data by prefecture-level city name.

### Measurement

#### Dependent variable: medical impoverishment

The dependent variable in this study is whether an individual's household is impoverished due to health payments (medical impoverishment). A non-poor household is impoverished by health payments when it becomes poor after paying for health services [1]. We follow the methodology proposed by WHO to calculate this variable as Eq. (1):

1 $$\left\{\begin{array}{c}I{mpoor}_{i,t}\\ I{mpoor}_{i,t}\end{array}\right.\begin{array}{c}=1, {exp}_{i,t}\ge {se}_{i,t}\ and\ {exp}_{i,t}-{oop}_{i,t}<{se}_{i,t}\\ =0, {exp}_{i,t}\ge {se}_{i,t}\ and\ {exp}_{i,t}-{oop}_{i,t}<{se}_{i,t}\end{array}$$

The medical impoverishment variable (Impoor_i,t_) is 1 when the household expenditure (${exp}_{i,t}$) is equal to or higher than the subsistence expenditure (${se}_{i,t}$) and the household expenditure net of out-of-pocket medical expenditure (${oop}_{i,t}$) is lower than the subsistence expenditure (${se}_{i,t}$); otherwise, it is 0.

The subsistence expenditure (${se}_{i,t}$) is calculated as Eq. (2). Subsistence expenditure (${se}_{i,t}$) is numerically equal to the poverty line (*pl*) multiplied by the equivalent household size (${eqsize}_{i,t}$), where the poverty line is the average monthly food expenditure of households whose food expenditure as a proportion of total household consumption expenditure is between the 45th and 55th percentile of the entire sample, and the equivalent household size is the 0.56th power of the household size [1].

2 $${se}_{i,t}=pl*{eqsize}_{i,t}$$

#### Core independent variable: urban–rural medical insurance integration

A binary variable was set according to whether the medical insurance integration policy was implemented in the respondent's city, where 1 indicates that the integration was implemented and 0 indicates that it was not.

#### Control variables

At the individual level, we controlled for characteristics that are often associated with the current status of an individual's life and thus affect whether the individual's household is impoverished, such as respondents' household registration, education, age, marital status, and personal income. For the household registration variable, the value of urban household registration is 0 and rural household registration is 1; for the education variable, the value of elementary school education and below is 1, junior high school is 2, high school is 3, and bachelor degree and above is 4; for the marital status variable, the value of no spouse (widowed, unmarried, divorced, cohabiting) is 0, otherwise it is 1. Besides, the age variable is the respondent's actual age value, and the personal income variable is the logarithm of the annual personal income.

In addition, we additionally control for city-level economic, demographic, and public health variables, specifically the log of hospital beds per capita, the log of GDP per capita, the log of average annual population, and the natural growth rate in the city where the individual is located.

### Data analysis

Our statistical analysis section is divided into 4 stages. In the first stage, we perform a descriptive statistical analysis of the data. In the second stage, we chose the difference-in-differences (DID) model with multiple time periods to conduct the baseline regression. To control for the effect of some unobservable variables on the results, we further included the individual fixed effects and year fixed effects in the DID model. The DID model is shown in Eq. (3).

3 $${MI}_{i,t}=a+{0integration}_{i,t}+{\beta X}_{i,t}+{\mu }_{i}+{\lambda }_{t}+{\epsilon }_{i,t}$$

In Eq. (3), ${MI}_{i,t}$ represents whether individual i experienced medical impoverishment at time t, and ${integration}_{i,t}$ represents whether the individual was in the treatment group at time t, taking the value of 1 if it was and 0 otherwise. ${X}_{i,t}$ is a vector of control variables. ${\mu }_{i}$ and ${\lambda }_{t}$ are individual fixed effects and year fixed effects, respectively. ${\epsilon }_{i,t}$ the error term.

In the third stage, we chose four types of robustness tests to demonstrate the robustness of the baseline regression results: (1) Parallel trend test [25]. The DID estimators are only meaningful when the parallel trend is satisfied, and we plot dynamic treatment effects to indicate the parallel trend. (2) PSM-DID. The PSM-DID method is able to eliminate some of the bias in the DID method, [26, 27] and we used the kernel matching method for the propensity score matching (PSM). (3) Treatment effect heterogeneity problem. Since the policy of integrating urban and rural medical insurance is not implemented at the same time nationwide, but by different cities themselves, there is some staggering of the policy implementation time. According to the idea proposed by Goodman-Bacon(2021), [28] this staggered DID will result in the ATT (average treatment effect on the treated) not being accurately estimated due to the heterogeneity of the treatment effects across cities. Therefore, using the doubly robust DID estimator proposed for this bias, [25] we re-estimate the ATT to show the robustness of our results. (4) Placebo test [29]. We advance the year of all policy implementation by 1 year to observe whether this pseudo-treatment would have the same effect on medical impoverishment.

Finally, in the fourth stage, we conducted population heterogeneity analysis by regressing subgroups of people with different demographic characteristics to explore the heterogeneity of policy effects on people with different characteristics. In addition, in order to reduce the existence of correlation between error terms and to obtain more robust estimates, we clustered the robust standard errors at the city level in the regression analyses described above.

The above statistical analysis process was done in STATA 16.0 software. *p* < 0.05 is the threshold of statistical significance.

## Result

### Descriptive statistics

Table 1 summarizes the descriptive statistics of the main variables. In the study sample, five percent of the individuals in the sample are in households that are impoverished because of health care costs. Besides, the average age of the sample is 43 years old, 88% of the population is married, the average educational level is junior high school, 75% samples are rural households, and the average annual personal income is 19,930 yuan (*e*^9.90^). Additionally, at the urban level, on average, the GDP per capita is 49,021 yuan (*e*^10.8^), the hospital beds per capita is 48 (*e*^3.88^), the annual population is 4.64 million (e^6.14^), and the natural growth rate is 7.73%.

**Table 1:** Descriptive statistics

	Mean (%)	Sd	Min	Max
Impoor	0.05	0.22	0.00	1.00
Integration	0.34	0.48	0.00	1.00
Age	42.56	11.39	15.00	64.00
Education	2.22	1.04	1.00	4.00
Marriage	88%	0.33	0.00	1.00
Ln_income	9.90	1.11	1.79	15.60
Household registration	75%	0.44	0.00	1.00
Ln_perGDP	10.80	0.59	9.08	11.89
Ln_perHBed	3.88	0.41	2.85	4.93
Ln_pop	6.14	0.66	4.67	8.13
Natural Growth Rate	7.73%	5.11	-8.90	30.30

### Baseline regression

Table 2 shows the results of the baseline regressions, the regression coefficient of the integration variable is negatively significant at the 1% significance level with or without the inclusion of control variables, suggesting that the urban–rural medical insurance integration policy can reduce the likelihood of medical impoverishment among residents.

**Table 2:** The results of the baseline regressions

	Medical impoverishment	
	(1)	(2)
Integration	-0.049^***^	-0.055^***^
	(0.01)	(0.02)
Control variables	No	Yes
Time FE	Yes	Yes
Individual FE	Yes	Yes
*N*	8397	8397

^***^ *p* < 0.05. The standard errors in parentheses are clustered by city. “YES” means the control variable or fixed effect was controlled, and “NO” means the control variable or fixed effect was not controlled

### Robustness tests

#### Parallel trend test

**Fig. 1:**
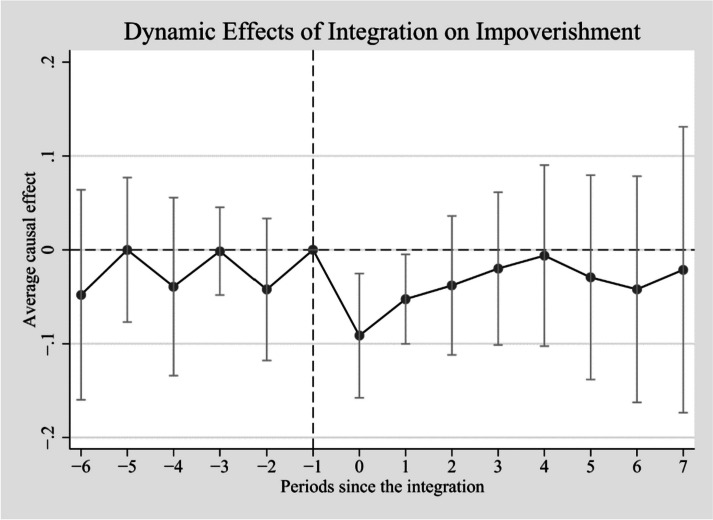
Parallel trend test. The standard errors in parentheses are clustered by city

Figure 1 illustrates the dynamic treatment effect, including the treatment effects and its 95% confidence intervals. The results show that there is no pre-trend before the urban–rural medical insurance integration, while there is a significant treatment effect after the onset of the policy, so our estimates satisfy the parallel trend assumption.

#### PSM-DID

Column (1) of Table 3 shows the results of the PSM-DID regression, where the regression coefficients are significantly negative at the 1% significance level and the treatment effect value is similar to the baseline regression results.

**Table 3:** Results of robustness tests

	PSM-DID	Robust DID	Placebo Test
	(1)	(2)	(3)
Integration	-0.051^***^	-0.045^***^	-0.008
	(0.01)	(0.02)	(0.02)
Control variables	Yes	Yes	Yes
Time FE	Yes	Yes	Yes
Individual FE	Yes	Yes	Yes
*N*	8397	6109	8397

^***^ *p* < 0.05. The standard errors in parentheses are clustered by city. “YES” means the control variable or fixed effect was controlled, and “NO” means the control variable or fixed effect was not controlled

#### Treatment effect heterogeneity

We re-estimated the ATT using a robust estimator [25] The results of the estimation are shown in column (2) of Table 3. The regression coefficient is -0.045 and significant at the 1% level, which is consistent with the baseline results.

#### Placebo test

Column (3) of Table 3 reports the results of the placebo test, and the nonsignificant estimated coefficients supporting the reliability of our baseline regression results.

### Heterogeneity analysis

#### Age

Table 4 shows the results of the age heterogeneity analysis, where the impact of the urban–rural medical insurance integration policy was concentrated on residents between the ages of 35 and 64, reducing the probability of medical impoverishment by 4.3% for residents aged 35–54 and 15.8% for residents aged 55–64.

**Table 4:** Results of age heterogeneity a nalysis

	(1)	(2)	(3)
	15–34	35–54	55–64
Integration	-0.020	-0.043^***^	-0.158^***^
	(0.03)	(0.02)	(0.05)
Control Variables	Yes	Yes	Yes
Time FE	Yes	Yes	Yes
Individual FE	Yes	Yes	Yes
*N*	1541	5061	1151

^***^ *p* < 0.05. The standard errors in parentheses are clustered by city. “YES” means the control variable or fixed effect was controlled, and “NO” means the control variable or fixed effect was not controlled

#### Household registration

The results in columns (1) to (4) of Table 5 show that the urban–rural medical insurance integration policy has a significant impact on the medical impoverishment of rural residents, but has no effect on residents with urban household. Specifically, the incidence of medical impoverishment among rural residents decreased by 6.7% after the implementation of the integration policy.

**Table 5:** Results of the analysis of household registration and migration heterogeneity

	Household Registration				Rural–Urban Migrated	
	(1)	(2)	(3)	(4)	(5)	(6)
	Urban	Urban	Rural	Rural	No	Yes
Integration	-0.006	-0.007	-0.060^***^	-0.067^***^	-0.071^***^	-0.086^***^
	(0.02)	(0.02)	(0.02)	(0.02)	(0.02)	(0.04)
Control Variables	No	Yes	No	Yes	Yes	Yes
Time FE	Yes	Yes	Yes	Yes	Yes	Yes
Individual FE	Yes	Yes	Yes	Yes	Yes	Yes
*N*	1760	1760	6148	6148	5884	207

^***^ *p* < 0.05. The standard errors in parentheses are clustered by city. “YES” means the control variable or fixed effect was controlled, and “NO” means the control variable or fixed effect was not controlled

#### Rural–urban migrant

Columns (5) and (6) of Table 5 give the regression results for the subsample of rural residents with and without rural–urban migration. The results show that the urban–rural medical insurance integration policy had a greater impact on the group that experienced migration, reducing their incidence of medical impoverishment by 8.6%, while only reducing the probability of medical impoverishment for those without migration by 7.1%.

## Discussion

Social medical insurance is an effective measure to provide financial protection to the population and is also considered a common policy tool to achieve universal health coverage in society [8]. This study examines the impact of urban–rural medical insurance integration on the medical impoverishment among Chinese resident households.

Overall, the integration policy reduces the incidence of medical impoverishment. This suggests that the integration policy not only equalizes medical insurance services between urban and rural areas, but also provides Chinese residents with stronger financial protection in health care by increasing medical insurance reimbursement rates and coverage, improving administrative efficiency, and consolidating risk pools, [29]. Thus, after the integration policy, China has successfully moved one step closer to its goal of achieving universal health coverage. This is consistent with existing research findings that integration policy will result in a lower probability of catastrophic health expenditures [30].

Also, by heterogeneity analysis, we find population heterogeneity in policy effects. First, in terms of age heterogeneity, residents will be increasingly likely to benefit from the integration policy as they get older. There are two possible explanations. On the one hand, the younger group is predictably unaffected because they are inherently healthy and have difficulty falling into diseases. On the other hand, older residents do exhibit a higher incidence of medical impoverishment, [20] so they are more likely to benefit from the integrated policy.

Second, in terms of household registration heterogeneity, we find that the policy effect of urban–rural medical insurance integration policy on medical impoverishment is mainly significant among rural residents, which may be caused by the fact that there is a large gap between the benefits of URBMI and NCMS before integration, while the urban–rural medical insurance integration policy allows rural residents to enjoy the same medical insurance benefits as urban residents.

Additionally, medical insurance integration would merge the administrative agencies and funding pools of URBMI and NCMS, enabling urban residents to benefit from administrative advances and pool risk reduction, [29] and some cities would also uniformly increase the reimbursement amount and scope of urban residents' medical insurance when implementing integration policies. Thus, the integration policies should have an impact on medical impoverishment for both urban and rural residents. However, our results found that the integration policy had no effect on the urban residents.

This may be due to the fact that urban residents are less likely to become poor due to illness than rural residents [17–20]. Under China's specific economic development conditions, urban residents are in a much better economic position than rural residents. In our study sample, the incidences of medical impoverishment were 1.4% among urban residents and 7.8% among rural residents, indicating the incidence of medical impoverishment is inherently much lower among urban than rural residents [17–20].

Another possibility is that the limited improvement of the reimbursement rate of urban residents after policy reform is insufficient to change the previous situation of poverty caused by illness. For example, in Beijing, the capital city of China, the inpatient reimbursement rate for urban residents has increased by 5–10%, while it has increased by more than 30% for some rural residents, [30] contributing to the fact that urban residents impoverished by illness benefit less from the policy.

Finally, in terms of the heterogeneity of urban–rural migration, those who move from rural to urban areas are more able to benefit from the policy than those who do not move. This may be largely due to the fact that prior to the integration, the NCMS was not available across counties, which meant that these residents who participated in the NCMS were not entitled to the medical insurance benefits even in the urban areas of the prefecture-level cities to which they belonged. After the integration, rural residents who are migrating to the city can enjoy the same medical insurance benefits as urban residents, without facing the problem of cross-location reimbursement procedures.

The contributions of this study are as follows: first, to our knowledge, this is the first study to identify the impact of urban–rural medical insurance integration on medical impoverishment through an econometric approach of multi-period DID at the city level. Second, although studies have shown the correlation between urban and rural health insurance integration policies and medical poverty, [31] they are based on cross-sectional data and therefore do not show the causal relationship between integration policies and medical poverty. In contrast, we utilized longitudinal survey data and a multi-period DID model at the prefectural city level, and conducted robustness tests such as parallel trend tests, hence, our estimation results were able to yield rigor and accuracy causality. Thirdly, we conducted heterogeneity analysis for different characteristics of the population to explore the differential impact of integration on various population samples, which allows us to provide a strong reference for the development of more targeted intervention policies.

It is worth noting that this study has certain limitations. First, because the data used in this study are from 2012 to 2016, we are unable to observe the effects of policy implementation after 2016. Future studies should focus on examining the policy effects of medical insurance integration after 2016, as well as the long-term effects in areas that have been integrated. Second, this study ignores samples that are already poor. Given the economic hardship of these samples themselves, they are likely to further exacerbate poverty after paying out-of-pocket health payments. Thus, it is likely that our study underestimates the impact of disease-based poverty or integration policy.

## Conclusion

In this study, we examined the impacts of urban–rural medical insurance integration on medical impoverishment in China. Our results show that the urban–rural medical insurance integration has been successful in reducing medical impoverishment, especially for older age, rural, and rural–urban migrated people. These conclusions can provide a reference for further improving social health insurance programs in China and also give suggestions for developing countries with similar social backgrounds to reduce medical impoverishment.

### Supplementary Information

**Additional file 1.** 

## Data Availability

Data will be made available on request.

## References

[1] World Health Organization. Distribution of health payments and catastrophic expenditures methodology. Geneva: World Health Organization; 2005. https://iris.who.int/bitstream/handle/10665/69030/EIP_?sequence=1.

[2] World Health Organization. The world health report: health systems financing: the path to universal coverage: executive summary. Geneva: World Health Organization; 2010. https://iris.who.int/bitstream/handle/10665/70496/WHO_IER_WHR_10.1_chi.pdf. 10.2471/BLT.10.078741 PMC2878164 20539847

[3] Liu Y, Rao K, Hsiao WC. Medical expenditure and rural impoverishment in China. J Health Popul Nutr 2003; 216–22. 14717567

[4] Devadasan N, Criel B, Van Damme W, Ranson K, Van der Stuyft P (2007). Indian community health insurance schemes provide partial protection against catastrophic health expenditure. BMC Health Serv Res.

[5] Mekonen AM, Gebregziabher MG, Teferra AS (2018). The effect of community based health insurance on catastrophic health expenditure in Northeast Ethiopia: a cross sectional study. PLoS One.

[6] Yardim MS, Cilingiroglu N, Yardim N (2010). Catastrophic health expenditure and impoverishment in Turkey. Health Policy.

[7] Zhang Y, Filipski MJ, Chen KZ (2019). Health insurance and medical impoverishment in rural China: evidence from Guizhou Province. Singap Econ Rev.

[8] Giedion U, Andrés Alfonso E, Díaz Y. The impact of universal coverage schemes in the developing world: a review of the existing evidence. 2013.

[9] Carrin G (2002). Social health insurance in developing countries: a continuing challenge. Int Soc Secur Rev.

[10] Gertler PJ (1998). On the road to social health insurance: the Asian experience. World Dev.

[11] Shaw RP (2007). Social health insurance for developing nations.

[12] Yu H. Universal health insurance coverage for 1.3 billion people: What accounts for China’s success? Health Policy. 2015; 119: 1145–52. 10.1016/j.healthpol.2015.07.008 PMC7114832 26251322

[13] Meng Q, Fang H, Liu X, Yuan B, Xu J (2015). Consolidating the social health insurance schemes in China: towards an equitable and efficient health system. Lancet.

[14] Yip WC-M, Hsiao WC, Chen W, Hu S, Ma J, Maynard A. Early appraisal of China’s huge and complex health-care reforms. Lancet. 2012; 379: 833–42. 10.1016/S0140-6736(11)61880-1 22386036

[15] Qiu P, Yang Y, Zhang J, Ma X (2011). Rural-to-urban migration and its implication for new cooperative medical scheme coverage and utilization in China. BMC Public Health.

[16] Meng Q, Xu K (2014). Progress and challenges of the rural cooperative medical scheme in China. Bull World Health Organ.

[17] Kumar K, Singh A, Kumar S (2015). Socio-economic differentials in impoverishment effects of out-of-pocket health expenditure in China and India: evidence from WHO SAGE. PLoS One.

[18] Li Y, Wu Q, Xu L (2012). Factors affecting catastrophic health expenditure and impoverishment from medical expenses in China: policy implications of universal health insurance. Bull World Health Organ.

[19] Li Y, Wu Q, Liu C (2014). Catastrophic health expenditure and rural household impoverishment in China: what role does the new cooperative health insurance scheme play?. PLoS One.

[20] Zhao Y, Oldenburg B, Mahal A, Lin Y, Tang S, Liu X (2020). Trends and socio-economic disparities in catastrophic health expenditure and health impoverishment in China: 2010 to 2016. Trop Med Int Health.

[21] Liu H, Zhao Z (2014). Does health insurance matter? Evidence from China’s urban resident basic medical insurance. J Comp Econ.

[22] Shi W, Chongsuvivatwong V, Geater A, Zhang J, Zhang H, Brombal D (2011). Effect of household and village characteristics on financial catastrophe and impoverishment due to health care spending in Western and Central Rural China: a multilevel analysis. Health Res Policy Syst.

[23] Qiu Z (2019). From “National Medical Insurance” to “Fair Medical Insurance”: current situation assessment and path analysis of the integration of medical insurance system for urban and rural residents in China. J Hebei Univ Philos Soc Sci.

[24] State Council. Opinions on the integration of the basic medical insurance system for urban and rural residents. 2016. http://www.gov.cn/zhengce/content/2016-01/12/content_10582.htm (Accessed 4 March 2023).

[25] Callaway B, Sant’Anna PH. Difference-in-differences with multiple time periods. J Econom. 2021; 225: 200–30.

[26] Imbens GW (2004). Nonparametric estimation of average treatment effects under exogeneity: a review. Rev Econ Stat.

[27] Sianesi B. An introduction to matching methods for causal inference and their implementation in Stata. 2010.

[28] Goodman-Bacon A (2021). Difference-in-differences with variation in treatment timing. J Econom.

[29] Huang X, Wu B (2020). Impact of urban-rural health insurance integration on health care: evidence from rural China. China Econ Rev.

[30] Li C, Xu L, Wang H, Wang X, Tang C (2020). The influence of integration of health-insurance schemes on catastrophic health expenditure in China: a cohort study. Lancet.

[31] Wang J, Zhu H, Liu H (2020). Can the reform of integrating health insurance reduce inequity in catastrophic health expenditure? Evidence from China. Int J Equity Health.

