# Supplementary material for: The impact of urban–rural medical insurance integration on medical impoverishment: evidence from China
Source: Int J Equity Health. 2023 Nov 23;22:245. doi: 10.1186/s12939-023-02063-6 (PMC10668423; doi:10.1186/s12939-023-02063-6)
Supplement: Supplementary file 1 — Additional file 1. [file 12939_2023_2063_MOESM1_ESM.docx]

**Supplemental Figure**

Figure S1: Spatial distribution of the cities implementing integration policy
